# Sensory Characteristics of Male Impala (*Aepyceros melampus*) Meat, Produced under Varying Production Systems and Nutrition

**DOI:** 10.3390/foods10030619

**Published:** 2021-03-15

**Authors:** Tersia Needham, Retha A. Engels, Louwrens C. Hoffman

**Affiliations:** 1 Department of Animal Science and Food Processing, Faculty of Tropical AgriSciences, Czech University of Life Sciences Prague, Kamýcká 129, 16500 Prague-Suchdol, Czech Republic; needham@ftz.czu.cz; 2 Department of Animal Sciences, University of Stellenbosch, Private Bag X1, Matieland, Stellenbosch 7602, South Africa; 17184495@sun.ac.za; 3 Center for Nutrition and Food Sciences, Queensland Alliance for Agriculture and Food Innovation (QAAFI), The University of Queensland, Health and Food Sciences Precinct, 39 Kessels Rd, Coopers Plains 4108, Australia

**Keywords:** fatty acids, game meat, *longissimus thoracis et lumborum*, flavor, tenderness

## Abstract

The objective of this study was to determine the influence of three production systems (intensive, semi-extensive and extensive) with differing nutrition on the descriptive sensory and fatty acid profiles of sub-adult (±15–18 months old) male impala *longissimus thoracis et lumborum* (LTL) muscles. The discriminant analysis plot showed that extensively produced impala had a sensory profile distinct from the intensive and semi-extensive system impala. Extensively produced impala had the highest sensory ratings for overall intensity, gamey, beef-like, herbaceous, and sweet-associated aroma and flavor of their meat. The intensive and semi-extensive system impala did not differ for most of the sensory attributes, except for higher ratings for gamey flavor, liver-like flavor, tenderness and mealiness, and lower ratings for residue found in semi-extensive system impala. The overall aroma and flavor intensities of impala meat in general had strong positive correlations with gamey, beef-like, herbaceous, and sweet-associated aromas and flavors; however, marketing should be adjusted depending on the nutrition received by the impala, to allow consumers to select their preferential sensory profile. Impala meat from all three production systems had low fat contents (<2%), and desirable fatty acid profiles.

## 1. Introduction

The South African game industry has expanded significantly in the last few decades [1], with legislation allowing the ownership of wildlife and the generation of revenues through sustainable activities being the key drivers [2,3,4]. Such activities include ecotourism, live sales, trophy hunting, meat (“biltong”) hunting and culling. These diverse activities, and the combined production of livestock in up to 50% of South African ranches [5], allows for greater financial stability in the wildlife industry [6]. Primarily, game ranching uses marginal land in South Africa, accounting for 14–17% of the country’s land surface area [1,7], and considering its position within the Biodiversity Economy Strategy [8], land conversion to wildlife ranching is likely to expand. Not only does game ranching contribute towards biodiversity goals in southern Africa, but it also aids in food security, with up to 48% of ranches in South Africa providing game meat to their employees [5]. Furthermore, they employ up to twice the number of people per unit area than livestock farms [5].

While meat and trophy hunting contribute approximately 41% of the revenue generated by game farms, the rising resistance towards hunting, particularly in social media [9], represents a real threat, as up to 50% of ranches depend on trophy hunting as their primary income [5]. On the other hand, commercial culling of game species currently contributes 3% of the financial revenue from game ranching in South Africa [5], representing a greatly underutilized meat source. The largest barriers in the integration being the different management objectives, high cost of compliance to current meat hygiene legislation, and higher effort to harvest wild species [5,10,11]. Thus, it is necessary to establish a well-regulated integration system of game meat into the commercial market that provides game ranchers with the incentive to increase game meat production [10]. Concurrently, it is necessary to understand consumer demands and acceptance of game meat products, to ensure that a high-quality product reaches the consumer, while developing premium products which are competitive against that of other livestock species.

In South Africa, up to 46% of game ranches currently use intensive breeding practices [5] to improve animal production and enable controlled breeding of high-value game species and coat color variants [12]. Together with the intensification of production systems, feed supplementation with total mixed rations (TMR) is often practiced to different extents to improve their breeding success and slaughter performance of culled animals [13]. The impala (*Aepyceros melampus*) is not only a popular choice for the breeding of color variants, such as the black impala [14], but it is one of most popular species for hunting, local consumption, and meat exportation [15]. Impala have a high fecundity [16], produce high carcass yields [17], and have tender meat [17,18], which makes them an ideal game animal to incorporate into the commercial meat market under sustainable culling regimes. Their meat is also low in intramuscular fat (IMF), even after feeding a TMR [17], which is currently considered desirable by consumers. This perception of high nutritional quality is also reliant on the fatty acid profile, with a high proportion of polyunsaturated fatty acids (PUFAs) and essential amino acids in meat being considered high quality [19]. On the other hand, saturated fatty acids (SFAs) have been related to the onset of atherosclerosis and consequently coronary vascular disease in humans [20,21], and thus it has been recommended by the World Health Organization (WHO) for people to increase their dietary PUFA:SFA ratio to above 0.4 [22]. The meat from various game species has been found to be high in PUFAs, with PUFA:SFA ratios above the recommended 0.4 and low n6:n3 fatty acid ratios [23], thus making it an appealing product for consumers. 

The sensory characteristics of meat plays an essential role in the satisfaction of consumers [11,19,24] and the consistency thereof remains imperative to consumer acceptability and repurchase of a meat product. However, the effects of diet on game meat flavor and aroma are poorly described [25]. While previous ruminant studies have focused primarily on the meat quality after grazing versus concentrate feeding, the feeding behavior of the impala is different to that of sheep and cattle, showing more mixed (browsing and grazing) feeding behavior depending on the vegetation present, and thus being influenced by season and region [12,16,26]. These differences in vegetation consumed influences the fatty acid profile of the meat, as shown where impala predominantly browsing on Mopani veld, (where *Colophospermum mopani* is the dominant tree species), differed from that of impala mostly grazing on grass in the Arid Sweet Bushveld [26]. Due to differences in the fatty acid profiles of animals consuming different vegetation, it is assumed that the flavor of game meat derived from various biomes in South Africa will be influenced [25]. 

However, the effect of feeding a TMR on the fatty acid profile and sensory meat quality of impala produced under intensified systems has not been quantified. This investigation is crucial, as feeding a concentrate diet high in grains is reported to influence the meat quality (in comparison to forage) in other ruminant species like sheep and cattle [27,28]. Such information on the fatty acid profile and sensory meat quality of impala is necessary for the commercial marketing of fresh products, product development, and consumer education regarding its nutritional quality. The aim of this study was thus to describe the physiochemical properties and sensory profiles of impala meat obtained from sub-adult males harvested within three different production systems (intensive, semi-extensive and extensive), utilizing three different production systems with differing nutritional conditions. 

## 2. Materials and Methods

A total of 36 sub-adult (±15–18 months old) male impala were obtained from two experimental locations in South Africa, namely Modimolle (Limpopo), in the central sandy bushveld bioregion of the Savanna biome, and Bredasdorp (Western Cape), in central rûens shale Renosterveld vegetation. The Renosterveld vegetation is characterized by grassy shrublands dominated by renosterbos (*Elytropappus rhinocerotis*), and includes shrubs of the *Aspalathus*, *Athanasia* and *Rhus* species [29]. The vegetation in the semi-extensive system (200-ha with 250–300 animals) in Modimolle included *Acacia*, *Euclea* and *Ziziphus* species, with *Burkea africana* and *Terminalia sericea* as prominent deciduous woodland species and *Panicum maximum* as a dominant grass species [17,18,29]. The intensive system in Modimolle utilized a 0.25-ha boma, in which 12 male impala were fed *ad libitum* with a commercial pelleted TMR (Table 1) for six months prior to slaughter. A boma is a small pen-like structure built to contain wild animals and allowing minimal movement, it has minimum vegetation, and all feed and water is supplied artificially. The nutritional content of the complete feed being 91.7% dry matter (8.3% moisture), 13.3% crude protein, 7.6% ash, 27.9% crude fiber, 47.7% neutral detergent fiber (NDF) and 30.5% acid detergent fiber (ADF), on an as-is basis [17,18]. Feed supplementation was provided in the semi-extensive system at a level of 800 g per animal per day (provided in the morning), with the same commercial feed as used in the intensive system, but the primary feed intake was the natural vegetation. A further 12 males were harvested from this semi-extensive system. The last 12 male impala were harvested from the extensive system in Bredasdorp, within an 800-ha camp. The only source of feed intake for the impala in the extensive system was the natural vegetation. 

### 2.1. Culling, Sample Collection and Chemical Analyses

All impala obtained for this study were culled during the day (ethical clearance number 10NP_HOF02) with suppressor-equipped light calibre rifles, and processed [17]. After the carcasses were cooled for 24 h at 4 °C, both the left and right *longissimus thoracis et lumborum* (LTL) muscles were excised, from the last rib caudally. The pH was measured in the right LTL, using a calibrated Crison pH25 m (Crison Instruments, Barcelona, Spain). The LTL muscles from the right carcass sides were trimmed of excess fat and connective tissue, pH was measured (Crison PH25 pH metre, Allela, Barcelona, Spain), and a section of each muscle was taken for training of the sensory panel and vacuum packaged. The remainder of the right LTL was weighed, and vacuum packaged for descriptive sensory analysis. The left LTL was vacuum packaged for chemical composition and fatty acid analysis. All samples were frozen at −20 °C until their respective analyses. The samples destined for sensory evaluation were frozen for two months, which is the standard export protocol during air shipment from southern African for game meat [30].

At the time of analyses, the left LTL muscles was thawed and homogenized. The moisture and ash contents were determined according to the Association of Official Analytical Chemist International [31]. The intramuscular fat (IMF) content was determined using a rapid solvent extraction [32], with chloroform/methanol, in a 1:2 ratio. Thereafter, the filtrate was dried at 60 °C and ground before nitrogen content analysis using a Leco Nitrogen/Protein Determinator (FP528, Leco Corporation). Crude protein content was calculated from the determined N-content using a conversion factor of 6.25.

### 2.2. Fatty Acid Analysis

Of the homogenized left LTL muscles, 1 g of each sample was used for fat extraction, using a chloroform:methanol (2:1; *v*/*v*) solution, with 0.01% butylated hydroxytoluene included as an anti-oxidant. Each sample was homogenized in the extraction solution with a polytron mixer (WiggenHauser, D-500 Homogenizer) for 30 s. For the quantification of each meat sample’s individual fatty acids, heptadecanoic acid (C17:0) was used as an internal standard (catalogue number H3500, Sigma-Aldrich, Gauteng, South Africa). A 250 μL sub-sample was collected from the extracted fat solution and transmethylated in a water bath set to 70 °C for two hours, with methanol:sulphuric acid (19:1; *v*/*v*) used as the transmethylating agent. Thereafter, the samples were removed from the water bath and allowed to cool at room temperature. Once cooled, water and hexane were used to extract the fatty acid methyl esters (FAMEs). Once the FAME-containing hexane solution and the distilled water were separated, the top hexane phase was transferred to a spotting tube to be dried in a water bath under nitrogen. After the sample had been dried, 50 μL of hexane was added to the spotting tube, which was then centrifuged. Of this final mixture, 1 μL was collected for injection into the gas chromatograph for analysis of separation FAMEs.

The FAMEs of each impala LTL sample were analyzed with a Thermo Scientific TRACE 1300 series gas-chromatograph (Thermo Electron Corporation, Milan, Italy) equipped with a flame-ionization detector (GC-FID), using a 30 m ZB-WAX Zebron 7HG-G007-11 capillary column with a 0.25 mm internal diameter and a 0.25 μm film thickness and a runtime of approximately 45 min. For each sample, 1 μL was injected in a 5:1 ratio, with helium used as the carrier gas (1 mL/min flow rate) and an injector temperature maintained at 260 °C. The oven temperature settings were as follows: initial temperature of 100 °C maintained for two minutes, followed by an increase at a rate of 10 °C per minute for four minutes until a 140 °C oven temperature was reached, after which the temperature was immediately increased by 3 °C per minute to reach 190 °C. This was followed by a final increase of 30 °C per minute, until the final temperature of 260 °C was reached, which was maintained for a minimum of five minutes. The FAME of each impala LTL sample was identified by comparing the retention times obtained to those of a standard FAME mixture (Supelco™ 37 Component FAME mix, Cat no. 47885-U, Supelco, Bellefonte, PA, USA) and quantified by comparing the integrated areas to those of the internal standard. The results are given as a percentage of the total FAME content. The cis and trans isomers of C18:1n9 and C18:2n6 are expressed as single combined values due to the co-elution of these isomers in most of the samples.

### 2.3. Descriptive Sensory and Shear Force Analyses

The impala meat was removed from the freezer and thawed at 4 ± 1 °C for 24 h prior to the pre-determined sensory analysis sessions. On the day of each session, each muscle sample was removed from its respective vacuum bag, blotted dry and weighed to determine thaw loss, as a percentage of the muscle weight before freezing. Thereafter, each sample was placed onto an aluminum foil-covered oven roasting pan and inserted into a separate oven bag. A thermocouple probe attached to a handheld digital temperature monitor (Hanna Instruments, Johannesburg, South Africa) was inserted into the middle of each meat sample and the prepared samples were placed in an oven preheated to 163 °C, and cooked until an internal temperature of 71 °C.

The meat samples were then removed from the oven and cooled for 10 min at room temperature, after which the samples were blotted dry and weighed to determine the cooking loss, as a percentage of the raw muscle weight. Each meat sample was cut into ±1.0 cm-thick steaks, which were the further cut into 1.0 cm^3^ meat cubes (excluding the outer cooked edges), and individually wrapped in aluminum foil. The wrapped meat cubes were then placed into ramekins (four cubes per ramekin) and re-heated for 10 min in a pre-heated oven at 100 °C. The ramekins containing the samples cubes were then transferred to water baths set at a temperature of 70 °C to maintain the heat of the meat samples for the duration of the training or testing session. Six rectangular cuboids (1 cm × 1 cm × 2 cm) were also taken for determining the Warner Bratzler shear force (WBSF) for each LTL, using an Instron Universal Testing Machine (Instron UTM, Model 2519-107, Instron^®^, Norwood, MA, USA) fitted with a Warner Bratzler blade (1 mm thick with a triangular opening, and 0.508 mm cutting edge radius; 5 kN capacity; 200 mm/min crosshead rate).

For the descriptive sensory analysis (DSA), a trained panel of 11 judges was used. The panelists were trained according to AMSA guidelines [33] and the consensus method. The training consisted of two session per day, for a total of four days. During these sessions, each judge received four meat cubes of each of the four reference samples (Table 2), as well as four meat cubes from each experimental animal from the three different production system treatments. The reference samples served as a baseline measurement and calibration of descriptors between the panelists, which allowed the panel to decide on a total of 23 sensory attributes, consisting of eight aroma attributes, 10 flavor attributes and five texture attributes (Table 3) to further evaluate the study samples. Once all panelists were confident with the agreed-upon descriptors, testing commenced. Testing consisted of two sessions per day for a duration of six days, with the 36 impala LTL muscles from the three different production systems randomly allocated into the 12 sessions. For the descriptive sensory analysis, the test re-test method was used [33]. The 11 panelists received the three production system treatments in a completely randomized order. Each panelist was allocated to a separate tasting booth equipped with computers, using Compusense^®^ five software (Compusense, Guelph, ON, Canada). The meat samples were rated by the panelists using an unstructured line scale ranging from zero to 100, according to Table 2, for each of the sensory attributes [33]. The DSA was held inside a temperature-controlled room (22 °C). Each panelist received distilled water, apple slices and biscuits to cleanse their palates between each meat sample.

### 2.4. Statistical Analyses

The data provided by the panelists for the descriptive sensory analysis was first evaluated with PanelCheck Software (Version 1.4.0, www.panelcheck.com, accessed in July 2017) to investigate the reliability of each panelist. No panelists were excluded, and data was analyzed using statistical software, version 9.4 (Stat 14.1, 2015, SAS Institute Inc., Cary, NC, USA). General linear models and univariate analysis of variances (ANOVAs) were used to evaluate the physical, chemical (fatty acids) and sensory meat quality data, where production system served as the treatment, and the LTL muscles of the impala were the replicates. The Shapiro–Wilk test was performed on the standardized residuals from the model to ensure normality, and no outlier values were identified or removed. Fisher’s least significant difference was the chosen post-hoc test for significant differences identified in the ANOVAs. Pearson’s correlation coefficients (r) were used to investigate the relationships between the physical, chemical, and sensory data obtained. The correlation matrixes can be found within the online supplementary material (Table S1). Associations between the sensory characteristics were illustrated by means of Principal Component Analysis (PCA) using XLSTAT^®^ (Version 2014.2.03; Addinsoft, New York, NY, USA). A significance level of 5% was used throughout.

## 3. Results

### 3.1. Physical Meat Quality

The influence of production system on the physiochemical properties of the impala LTL meat is presented in Table 4. Production system influenced the chemical composition of the LTL for moisture (*p* < 0.001), protein (*p* < 0.001), and IMF (*p* < 0.001) contents. The intensively produced impala fed the TMR *ad libitum* had greater IMF percentages and lower crude protein percentages than impala produced extensively without any supplementary feeding. Impala from the extensive production system produced meat with lower thaw losses, and higher pH, than impala from the intensive or semi-extensive production systems. No differences were found between the impala meat from the different production systems for cooking loss percentage. Shear force values after grilling the meat from the three different production systems were only bordering significance (Table 4).

### 3.2. Fatty Acids

The fatty acid profile of the impala meat from the three different production systems showed only minor differences (Table 5). The impala that received some concentrate feeding (intensive and semi-extensive) had greater saturated fatty acids (SFA) percentages than the extensively produced impala meat (*p* = 0.016), but no differences in monounsaturated fatty acid (MUFA) and total polyunsaturated fatty acid (PUFA) contents were found. Regarding the specific fatty acids, intensive system impala meat had greater lignoceric acid (C24:0; *p* = 0.009) lauric acid (C12:0; *p* = 0.043), eicosatrienoic (C20:3*n*3; *p* = 0.001) contents, and lower cis-10-pentadecenoic (C15:1*n*9t; *p* = 0.048), alpha-linolenic (C18:3*n*3; *p* < 0.001) contents, than the meat from the extensive system. However, only the lignoceric, and alpha-linolenic acid contents of the intensive system impala meat differed from the semi-extensive system impala meat (Table 5).

### 3.3. Descriptory Sensory Characteristics

Production system had an influence on all the sensory characteristics (Table 6) except liver-like aroma, salty taste, initial juiciness, and sustained juiciness. Impala from the extensive production system produced meat with the highest overall aroma and overall flavor intensities. Intensive and semi-extensive system impala meat did not differ significantly from each other for overall aroma intensity. However, the lowest overall flavor intensity was found in impala from the intensive production system, while semi-extensive system impala did not differ significantly from either of the other two systems (Table 6).

Extensively produced impala had a significantly higher intensity for gamey aroma, beef-like aroma and flavor, herbaceous aroma and flavor, sweet-associated aroma and taste, and lower metallic aroma, metallic flavor and off, sour, sweat-like flavors than the other two systems’ impala meat, which did not differ from each other for these characteristics. Gamey flavor was found to have the lowest (*p* < 0.001) intensity in meat from intensive system impala meat compared to that of semi-extensive and extensive system impala, while the latter two systems did not differ significantly from each other. While the liver-like aroma did not differ between production systems, liver-like flavor had a higher (*p* = 0.002) intensity in semi-extensive system impala than in intensive or extensive system impala meat, which did not differ from each other. Semi-extensive system impala meat also had the highest (*p* = 0.041) intensity for sour taste, while the lowest sour taste intensity was found in extensive system impala meat (but both were no different to the intensive system impala meat). In terms of texture attributes, the most tender (*p* = 0.016) meat was found in semi-extensive system impala meat, while the intensive and extensive system impala did not differ from one other. Impala from the semi-extensive system also produced meat with the lowest residue and highest mealiness scores, while the other two systems did not differ significantly from each other for these characteristics (Table 6). 

**Figure 1:**
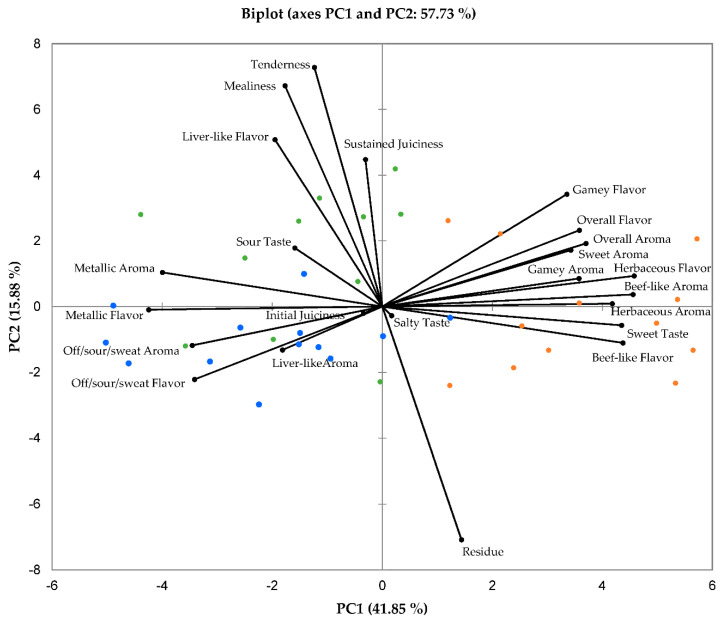
Principal component analysis (PCA) biplot depicting the means of the sensory attributes of sub-adult male impala *longissimus thoracis et lumborum* meat. A total mixed ration was fed in the intensive system (*n* = 12), while supplementation (with the same ration) was provided on Savanna vegetation (*n* = 12), and no supplementation was provided to the extensive system on Renosterveld vegetation (*n* = 12). The intensive system impala meat samples are represented blue, the semi-extensive system impala meat samples in green and the extensive system impala meat samples in orange.

The PCA biplot in Figure 1 illustrates the relationships between the various sensory characteristics of the impala meat evaluated. The combination of PC1 and PC2 explained 57.7% of the total variance, of which 41.9% is explained by PC1, and 15.9% by PC2. It can be observed that the extensive production system impala meat is associated with the sensory characteristics on the right side of PC1, while both intensive and semi-extensive system impala show stronger associations with the attributes on the left side of PC1. The intensive and semi-extensive treatments also show a substantial amount of overlap for the variables on the left of F1, whereas the extensive production system shows very little overlap with either of the other two treatments.

### 3.4. Correlations between Physical, Chemical, and Sensory Meat Characteristics

Of the extensive correlations shown in Table S1, Supplementary Material, the most noteworthy are highlighted here. A strong negative correlation existed (r = −0.772; *p* < 0.001) between the pH values and the thaw loss percentages obtained for impala during the sensory trial (Table S1). However, no correlations were found between initial or sustained juiciness and thaw loss, or between either of the juiciness sensory attributes and the pH of the impala meat (Table S1). A strong negative correlation (*r* = −0.745; *p* < 0.001) was also found between the WBSF values and the tenderness sensory rating of the impala meat. Additionally, strong correlations were found between sensory tenderness and residue (*r* = −0.916; *p* < 0.001), as well as mealiness (*r* = 0.838; *p* < 0.001). Strong positive correlations (*p* < 0.001) found between overall intensity and the gamey and beef-like attributes for aroma and flavor (Table S1). Furthermore, strong positive correlations (*p* < 0.005) were found between the aroma and flavor of the overall intensity, gamey, beef-like, metallic, herbaceous, sweet-associated, and off, sour, sweat-like sensory attributes (Table S1). Alpha-linolenic acid showed strong correlations with herbaceous aromas and flavors (*r* = 0.556; *p* < 0.001 and *r* = 0.477; *p* = 0.003, respectively). Positive correlations were also found between C18:3n3 and gamey aroma and flavor (Table S1), as well as between liver-like flavors and the C18:1n9c (oleic) fatty acid (*r* = 0.338; *p* = 0.044). Eicosatrienoic (C20:3n3) acid was found to have a negative correlation (*r* = −0.401; *p* = 0.015) to alpha-linoleic acid, and therefore impala with lower alpha-linolenic acid contents will have higher eicosatrienoic acid contents, as may be observed with intensive system impala (Table 4). A negative correlation (*r* = −0.352; *p* = 0.035) was found between gamey flavor and total IMF content of impala meat, thus indicating that the intensity of gamey flavor will increase as the IMF content decreases. The IMF content and SFA content of impala meat were positively correlated (*r* = 0.938; *p* < 0.001). 

## 4. Discussion

The differences in proximate composition, fatty acid profile and physical meat quality parameters of the meat from the male impala kept under different productions systems and nutrition was minor. The greatest difference in this regard was seen in the thaw loss, where meat produced under extensive conditions had almost 50% less moisture loss after thawing. This parameter is particularly important for game meat, as when exportation is possible, the meat is typically transported frozen. The freezing and subsequent thawing of meat influences the distribution and content of moisture within the meat, and consequently the amount of moisture lost as exudate (thaw loss) [34], which can influence consumers’ willingness to purchase the product. Thus, lower thaw losses may be beneficial for the visual presentation of frozen (or pre-frozen) impala meat. While lower thaw losses may indicate dark, firm, dry (DFD) meat, which is often seen in extensively harvested game species [35], the tenderness indicators (shear force and sensory scores) did not indicate DFD conditions in the extensively produced impala meat. 

Meat tenderness is one of the most important parameters of meat quality for consumers, and influences consumer experience and acceptance [36]. In the present study, shear force values were, as expected, strongly negatively correlated with sensory tenderness ratings of the impala meat. The semi-extensive impala meat showed the highest tenderness scores, thus producing more tender meat compared to that of the other two systems. The strong correlations between tenderness scores, residue and mealiness explains why the meat of semi-extensive system impala were ranked lower for residue and higher for mealiness. While mealiness is often considered to be a negative characteristic, the mealiness ratings of impala from all three production systems was actually low (< 10), indicating that the there was little disintegration of muscle fibers into small particles (within first few chews). In addition to textural sensory characteristics, consumer satisfaction regarding meat is driven by aroma and flavor [37]. 

Game species have a characteristic aroma and flavor, that is distinct from that of domestic livestock [38], with “gamey” attributes specifically defined for game meat as an aroma or flavor associated with meat from wild animal species [25]. However, consumer acceptance of cooked meat has been positively correlated to higher beef-like aroma and flavors, as well as a sweet-associated aroma and taste [39], and negatively correlated to gamey, metallic, and liver-like aromas and flavors, as well as sour taste, residue and mealiness [38]. Production system had a significant influence on most of the sensory attributes evaluated in the impala meat, with the extensive system impala meat being distinctive from the impala fed supplementary feed or fed entirely on a TMR. Despite having no access to natural vegetation for six months prior to slaughter, the TMR-fed impala in the intensive system had similar aroma and flavor scores, except for gamey and liver-like flavors, where the intensively produced impala meat showed lower (and thus improved) scores. Thus, whether the animals consumed natural Savanna vegetation or not appears to affect the “gaminess” and “liver-like” flavor of impala meat, and thus feeding a TMR for six months prior to slaughter may improve consumer acceptability of impala meat from this region. On the other hand, the impala from the extensive system, grazing so-called fynbos vegetation in the Renosterveld biome of South Africa, had higher scores for herbaceous, beef-like, gamey, and sweet-associated aromas and flavors, and lower for off, sour, sweat-like, metallic, and liver-like aromas and flavors. In comparison to the impala fed with concentrate feed (intensively and semi-extensively), the impala eating the Renosterveld scored higher for overall aroma and flavor intensity, likely due to the vegetation present in the area. Thus, the sensory profile of the impala meat produced extensively in the Renosterveld region of the Western Cape may be regarded as unique relative to that of impala meat from the other two production systems. This contrasts with studies evaluating the consumer acceptance of pasture-versus grain-fed sheep, where lambs raised on grain-based diets only, or supplemented with grain-based diets on pasture, had greater sensory acceptability than pasture or forage-raised lamb [40,41]. The reasons for these differences in sensory profiles in the lamb being attributed to differences in fat (subcutaneous and intramuscular) and its fatty acid profile [42]. However, it is difficult to compare such studies of traditional livestock meat (like lamb and beef) with game meat, as their vegetation consumption differs due to their production region and their different feeding strategies.

In studies on reindeer (*Rangifer tarandus*) and red deer (*Cervus elaphus*), it was found that animals finished on grazing produced meat with higher ratings for gamey flavor, and sweet taste, compared to deer finished on pellets. These differences in flavor were thought to be the consequence of natural grazing and partially due to differences in fatty acid compositions of the meat resulting from differences in the dietary regimes of the animals [38,43]. Similarly, impala finished on dietary regimes with higher grass contents have been found to produce meat with higher contents of C18:3*n*3 (alpha-linolenic) fatty acid [26], as this PUFA is naturally found in high concentrations in grasses and plant leaves. In the present study, the highest alpha-linolenic acid content was found in semi-extensive and extensive production system impala meat, with large amounts of grazing and browsing material forming part of the natural vegetation of both systems. Alpha-linolenic acid concentration was correlated with herbaceous aromas and flavors, which may explain these distinct organoleptic attributes in the extensively produced impala meat, as it also had (numerically) the highest alpha-linolenic acid concentrations. This is not surprising, as the natural vegetation in the Renosterveld region, being fynbos, is also high in alpha-linolenic acid. Alpha-linolenic acid is an important n3 essential fatty acid for the health of humans, and regular consumption would be beneficial due to the proactive influence of this fatty acid on cardiovascular deterioration [20]. The lower alpha-linolenic acid content in the meat from intensive system impala may be related to their TMR, as fallow deer fed a concentrate-based diet have lower PUFA concentrations in their meat than those grazing on pasture [44]. As expected, feeding the concentrated diet increased the SFA content of the impala meat in the present study, as also seen in other ruminant species when fed high energy diets [40]. Meat with lower SFA contents is often considered more desirable by health-conscious consumers, while unsaturated fatty acids (MUFAs and PUFAs) are also considered to be desirable fatty acids as they decrease the levels of low-density lipoprotein cholesterol in the blood [21].

While differences in sensory profiles of grain-versus grass-fed lamb have also been primarily attributed to differences in fatty acid profiles [45], in the case of natural South African vegetation, other compounds may influence the sensory profile of extensively raised lamb. Extensively produced South African Dorper lambs, which consume a diet high in aromatic Karoo bushes and shrubs (found in Northern parts of South Africa), produce meat with distinctive herbaceous sensory characteristics. The unique herbaceous characteristics of meat from Karoo lambs were found to be caused by a high concentration of terpenes in the Karoo vegetation, which are volatile compounds that often have a strong aroma [46]. Considering the low IMF content of the impala meat in the present study, similar volatile compounds may affect the sensory profile of the extensively produced impala on fynbos vegetation, and should be investigated further. Like “Karoo lamb” in South Africa, the registration and marketing of the meat from impala fed Renosterveld vegetation could also be considered, allowing consumers to make informed decisions regarding their selection either for or against these qualities.

## 5. Conclusions

The sensory meat quality of impala was significantly affected by the different nutritional conditions within the three different production system. Extensively produced impala eating Renosterveld/Fynbos vegetation had meat with a sensory profile that was distinct from the other two systems, which was primarily “herbaceous”, likely caused by the fragrant Fynbos vegetation in their diet. Feeding only a TMR diet to the impala in the Savanna biome decreased the “gamey” and “liver-like” attributes of their meat, which may be preferred by consumers sensitive to this flavor attribute but seeking a “healthier” alternative to beef and lamb. Despite differences in IMF content, the fatty acid profile of all the impala meat did not show any major differences, and thus the meat from all systems may be marketed as a healthy lean meat alternative to traditional “red” meat species. However, future marketing should also consider distinguishing between impala meat products from different production regions. 

## Figures and Tables

**Table 1:** Feed ingredients and inclusion rates for the total mixed ration fed to male impala on an *ad libitum* basis in the intensive production system.

Feed Ingredient	Inclusion (%)
Lucerne meal	38.7
Maize	16.1
Molasses meal	12.9
Sunflower oilcake meal	9.7
Eragrostis hay	8.1
Melagac rumen bypass fat	1.6
Vitamins and mineral premix (Game Production Macropack)	12.9

**Table 2:** Reference samples used during the training phase for the descriptive sensory analysis (DSA) of impala meat, by a panel of 11 judges.

Sample	Reference Purpose	Internal Cooking Temperature	Scale
Beef fillet	Beef-like aroma and flavor, initial and sustained juiciness, tenderness, residue, mealiness	71 °C	0 = low intensity; 100 = high intensity
Beef ox liver	Liver-like aroma and flavor	No probe	
Beef rib-eye	Initial juiciness, sustained juiciness, toughness, residue, mealiness	72 °C	
Ostrich fillet	Metallic aroma and flavor	71 °C	

**Table 3:** Description and scale of the sensory attributes (aroma, flavor, and texture) decided upon by the trained sensory panel for the descriptive sensory analysis (DSA) of impala meat (*longissimus thoracis et lumborum*) from three different production systems.

Sensory Attribute	Description of Attributes	Scale
**Aroma and Flavor**		
Overall intensity ^a^	Intensity of aroma in the first few sniffs and the intensity of all flavors.	0 = low intensity; 100 = high intensity
Gamey ^a^	Aroma/flavor associated with meat from wild animal species.	
Beef-like ^a^	Aroma/flavor associated with cooked beef fillet.	
Metallic ^a^	Aroma/flavor associated with raw meat/blood-like aroma/flavor.	
Liver-like ^a^	Aroma/flavor associated with pan-fried beef liver.	
Herbaceous ^a^	Aroma/flavor associated with earthy, Fynbos-like herbs.	
Off/sour/sweat-like ^a^	Aroma/flavor associated with an off/sour/sweat-like characteristic of meat.	
Sweet-associated aroma	Aroma associated with the browning of meat (Maillard reaction).	
Sweet-associated taste	Taste associated with a sucrose solution.	
Salty taste	Taste associated with sodium ions.	
Sour taste	Taste associated with a citric acid solution.	
**Texture**		
Initial juiciness	Amount of fluid extruded when pressed perpendicular to fibres, between the thumb and forefinger.	0 = dry; 100 = extremely juicy
Sustained juiciness	Amount of moisture perceived during mastication (after five chews).	0 = dry; 100 = extremely juicy
Tenderness	Impression of tenderness after mastication (after five chews).	0 = tough; 100 = extremely tender
Residue	Residual tissue remaining after mastication (after 10 chews).	0 = none; 100 = abundant
Mealiness	Disintegration of muscle fibers into small particles (within first few chews).	0 = none; 100 = abundant

^a^ Sensory attribute was evaluated for both aroma and flavor.

**Table 4:** LSMeans and the pooled standard error (SEM) of the chemical and physical parameters of sub-adult male impala *longissimus thoracis et lumborum* meat as influenced by production system. A total mixed ration was fed in the intensive system (*n* = 12), while supplementation (with the same ration) was provided on Savanna vegetation (*n* = 12), and no supplementation was provided to the extensive system on Renosterveld vegetation (*n* = 12).

Parameter	Production System			SEM	*p*-Value
	Intensive	Semi-Extensive	Extensive		
**Chemical composition (g/100 g meat)**					
Moisture	75.3 ^b^	75.7 ^a^	74.7 ^c^	0.10	<0.001
Crude Protein	22.7 ^b^	22.0 ^c^	23.4 ^a^	0.12	<0.001
Intramuscular fat	2.0 ^a^	1.8 ^b^	1.5 ^c^	0.05	<0.001
Ash	1.21	1.19	1.18	0.01	0.062
**Physical meat quality**					
pH 24 h post-mortem	5.8 ^b^	5.6 ^c^	6.2 ^a^	0.05	<0.001
Thaw loss (%)	9.9 ^a^	10.1 ^a^	4.0 ^b^	0.50	<0.001
Cooking loss (%)	29.9	29.3	30.5	2.09	0.921
Shear force (N)	52.5	37.2	52.3	5.13	0.068

^a,b,c^ Means with different superscripts within a parameter differ significantly from each other (*p* ≤ 0.05).

**Table 5:** LSMeans and the pooled standard error (SEM) of the fatty acid profiles of sub-adult male impala *longissimus thoracis et lumborum* meat as influenced by production system. A total mixed ration was fed in the intensive system (*n* = 12), while supplementation (with the same ration) was provided on Savanna vegetation (*n* = 12), and no supplementation was provided to the extensive system on Renosterveld vegetation (*n* = 12).

Parameter	Production System			SEM	*p*-Value
	Intensive	Semi-Extensive	Extensive		
Total fatty acids (mg/g meat)	19.7	17.8	17.6	0.88	0.181
**Fatty Acids (% of total FAMEs):**					
C6:0 (Hexanoic)	2.5	2.3	2.3	0.07	0.061
C8:0 (Caprylic)	2.5	2.5	2.3	0.09	0.416
C10:0 (Capic)	2.6	2.5	2.4	0.11	0.634
C12:0 (Lauric)	2.7 ^a^	2.8 ^a^	2.4 ^b^	0.12	0.043
C14:0 (Myristic)	3.0 ^a^	3.0 ^a^	2.7 ^b^	0.10	0.027
C15:0 (Pentadecyclic)	1.1	1.1	1.0	0.13	0.722
C16:0 (Palmitic)	10.0 ^b^	11.8 ^a^	9.4 ^b^	0.48	0.003
C18:0 (Stearic)	12.3	12.4	12.3	0.35	0.967
C20:0 (Arachidic)	1.2	1.2	1.1	0.04	0.127
C22:0 (Behenic)	1.9	2.1	2.3	0.12	0.089
C24:0 (Lignoceric)	2.9 ^a^	2.5 ^b^	2.5 ^b^	0.10	0.009
**Total SFA (% of total FAMEs)**	42.7 ^ab^	44.2 ^a^	40.7 ^b^	0.82	0.016
C14:1n9c (Myristoleic)	1.1	1.1	1.00	0.04	0.146
C15:1n9t (Cis-10-pentadecenoic)	1.7 ^b^	1.8 ^b^	2.6 ^a^	0.27	0.048
C16:1*n*7 (Palmitoleic)	1.7	1.8	1.6	0.06	0.154
C17:1 (Heptadecenoic)	1.7	1.6	1.5	0.09	0.237
C18:1*n*9 (Oleic)	5.0	6.0	5.8	0.44	0.307
C20:1*n*9 (Gondoic)	1.0	1.1	1.0	0.04	0.444
**Total MUFA (% of total FAMEs)**	12.3	13.3	13.5	0.56	0.269
C18:2*n*6 (Linoleic)	7.5	7.7	7.8	0.22	0.687
C18:3*n*6 (Gamma-linolenic)	4.5	4.4	4.2	0.15	0.202
C18:3*n*3 (Alpha-linolenic)	1.5 ^b^	2.6 ^a^	3.0 ^a^	0.14	< 0.001
C20:2n6 (Eicosadienoic)	4	2.6	3.8	0.73	0.325
C20:3*n*6 (Dihomo-gamma-linolenic)	2.0	2.0	1.9	0.07	0.555
C20:3*n*3 (Eicosatrienoic)	10.0 ^a^	8.3 ^b^	7.8 ^b^	0.39	0.001
C20:5*n*3 (Eicosapentaienoic)	2.4 ^b^	2.9 ^ab^	4.6 ^a^	0.61	0.045
C22:2*n*6 (Docosadienoic)	4.5	3.5	4.2	0.41	0.239
C22:6*n*3 (Docosahexaenoic)	7.5	7.5	7.5	0.24	0.989
**Total PUFA (% of total FAMEs)**	45	42.5	45.8	1.28	0.176
PUFA:SFA ratio	1.1	1.0	1.1	0.05	0.098
n6 PUFA	23.7	21.2	23	0.99	0.201
n3 PUFA	21.4	21.3	22.9	0.56	0.102
n6:n3 PUFA ratio	1.1	1.0	1.0	0.05	0.144

^a,b^ Means with different superscripts in the same row differ significantly (*p* ≤ 0.05) from each other. Abbreviations: SFA = Saturated fatty acids (includes C6:0, C8:0, C10:0, C12:0, C14:0, C15:0, C16:0, C18:0, C20:0, C22:0 and C24:0); MUFA = Monounsaturated fatty acids (includes C14:1*n*9c, C15:1*n*9t, C16:1n7, C17:1, C18:1*n*9c and C20:1*n*9); PUFA = Polyunsaturated fatty acids (includes C18:2*n*6c, C18:3*n*6, C18:3*n*3, C20:2*n*6, C20:3*n*6, C20:3*n*3, C20:5*n*3, C22:2*n*6 and C22:6*n*3).

**Table 6:** LSMeans and the pooled standard error (SEM) of the sensory scores of sub-adult male impala *longissimus thoracis et lumborum* meat, as influenced by production system. A total mixed ration was fed in the intensive system (*n* = 12), while supplementation (with the same ration) was provided on Savanna vegetation (*n* = 12), and no supplementation was provided to the extensive system on Renosterveld vegetation (*n* = 12).

Sensory Characteristic	Production System			SEM	*p*-Value
	Intensive	Semi-Extensive	Extensive		
**Aroma**					
Overall aroma intensity	65.1 ^b^	66.3 ^b^	69.1 ^a^	0.49	<0.001
Gamey aroma	54.7 ^b^	56.1 ^b^	58.5 ^a^	0.59	<0.001
Beef-like aroma	37.2 ^b^	38.5 ^b^	42.4 ^a^	0.53	<0.001
Metallic aroma	6.3 ^a^	6.0 ^a^	2.4 ^b^	0.56	<0.001
Liver-like aroma	1.8	2.2	1.5	0.35	0.461
Herbaceous aroma	6.8 ^b^	8.0 ^b^	13.2 ^a^	0.52	<0.001
Off, sour, sweat-like aroma	5.5 ^a^	3.6 ^ab^	2.4 ^b^	0.69	0.014
Sweet-associated aroma	8.4 ^b^	9.5 ^b^	11.5 ^a^	0.42	<0.001
**Flavor**					
Overall flavor intensity	62.9 ^b^	64.2 ^ab^	65.7 ^a^	0.61	0.008
Gamey flavor	54.0 ^b^	55.9 ^a^	56.7 ^a^	0.42	<0.001
Beef-like flavor	39.4 ^b^	38.5 ^b^	45.0 ^a^	0.66	<0.001
Metallic flavor	8.4 ^a^	8.4 ^a^	3.3 ^b^	0.51	<0.001
Liver-like flavor	1.2 ^b^	2.2 ^a^	0.6 ^b^	0.30	0.002
Herbaceous flavor	7.1 ^b^	8.2 ^b^	12.1 ^a^	0.48	<0.001
Off, sour, sweat-like flavor	1.3 ^a^	0.9 ^a^	0.2 ^b^	0.24	0.012
Sweet-associated taste	10.5 ^b^	10.2 ^b^	12.6 ^a^	0.32	<0.001
Salty taste	9.1	9.1	9.1	0.01	1.000
Sour taste	4.2 ^ab^	4.5 ^a^	3.7 ^b^	0.22	0.041
**Texture**					
Initial juiciness	39.4	39.3	39.7	0.98	0.950
Sustained juiciness	46	45.7	46.5	0.83	0.772
Tenderness	59.9 ^b^	66.9 ^a^	59.7 ^b^	1.90	0.016
Residue	11.3 ^a^	6.9 ^b^	12.2 ^a^	1.31	0.015
Mealiness	6.0 ^b^	9.9 ^a^	4.7 ^b^	0.85	<0.001

^a,b^ Means with different superscripts in the same row differ from one another (*p* ≤ 0.05).

## Data Availability

The data presented in this study are available on request from the corresponding author.

## Supplementary Materials

The following are available online at https://www.mdpi.com/2304-8158/10/3/619/s1, Table S1 shows the correlation matrix between the physical, chemical, and sensory meat characteristics for the impala meat for all the three production systems pooled together.
