# Supplementary material for: Sensory Characteristics of Male Impala (Aepyceros melampus) Meat, Produced under Varying Production Systems and Nutrition
Source: Foods. 2021 Mar 15;10(3):619. doi: 10.3390/foods10030619 (PMC8001185; doi:10.3390/foods10030619)
Supplement: Supplementary file 1 [file foods-10-00619-s001.pdf]

Sensory characteristics of male impala (*Aepyceros melampus*) meat, produced under varying production systems and nutrition

Tersia Needham [1,2], Retha A. Engels [2] and Louwrens C. Hoffman [2,3*]

[1] Department of Animal Science and Food Processing, Faculty of Tropical AgriSciences, Czech University of Life Sciences Prague, Kamýcká 129, 16500 Prague-Suchdol, Czech Republic; needham@ftz.czu.cz
[2] Department of Animal Sciences, University of Stellenbosch, Private Bag X1, Matieland, Stellenbosch 7602, South Africa; retha.engels@meadownatal.co.za
[3] Center for Nutrition and Food Sciences, Queensland Alliance for Agriculture and Food Innovation (QAAFI), The University of Queensland, Health and Food Sciences Precinct, 39 Kessels Rd, Coopers Plains 4108, Australia
* Correspondence: louwrens.hoffman@uq.edu.au; Tel.: +61-4-1798-4547

Supplementary Material

Table S1 Correlation matrix for the Pearson correlation coefficients (*r*) for the sensory characteristics and fatty acids obtained in impala meat. Values in bold are significant at a level of $P \leq 0.05$.

Variable	1	2	3	4	5	6	7	8	9	10	11	12
1	**1**	**<0.001**	**<0.001**	**0.001**	0.982	**<0.001**	**0.005**	**<0.001**	**<0.001**	**0.000**	**0.000**	**<0.001**
2	**0.782**	**1**	**<0.001**	**0.001**	0.872	**<0.001**	**0.004**	**0.001**	**0.000**	**0.004**	**0.008**	**<0.001**
3	**0.679**	**0.693**	**1**	**<0.001**	0.061	**<0.001**	**<0.001**	**<0.001**	**<0.001**	**<0.001**	**<0.001**	**<0.001**
4	**-0.549**	**-0.517**	**-0.736**	**1**	**0.031**	**<0.001**	**0.006**	**0.001**	**0.009**	**0.000**	**<0.001**	**<0.001**
5	-0.004	-0.028	-0.315	**0.360**	**1**	0.259	0.192	0.635	0.231	**0.004**	**0.030**	**0.019**
6	**0.751**	**0.626**	**0.744**	**-0.691**	-0.193	**1**	**0.001**	**<0.001**	**0.001**	**0.001**	**<0.001**	**<0.001**
7	**-0.457**	**-0.467**	**-0.739**	**0.447**	0.222	**-0.513**	**1**	**0.001**	**<0.001**	**0.000**	**0.001**	**0.003**
8	**0.614**	**0.521**	**0.618**	**-0.540**	-0.082	**0.712**	**-0.551**	**1**	**0.001**	**0.005**	**0.000**	**0.000**
9	**0.603**	**0.567**	**0.716**	**-0.429**	-0.205	**0.515**	**-0.687**	**0.526**	**1**	**<0.001**	**<0.001**	**0.004**
10	**0.552**	**0.473**	**0.604**	**-0.554**	**-0.473**	**0.548**	**-0.567**	**0.461**	**0.680**	**1**	**0.001**	**0.003**
11	**0.580**	**0.432**	**0.781**	**-0.717**	**-0.362**	**0.697**	**-0.525**	**0.602**	**0.652**	**0.550**	**1**	**<0.001**
12	**-0.662**	**-0.622**	**-0.794**	**0.760**	**0.388**	**-0.686**	**0.481**	**-0.578**	**-0.468**	**-0.486**	**-0.742**	**1**
13	-0.198	-0.162	**-0.341**	0.284	-0.177	**-0.349**	0.186	-0.278	-0.218	0.127	**-0.468**	0.322
14	**0.737**	**0.689**	**0.866**	**-0.738**	**-0.333**	**0.818**	**-0.555**	**0.688**	**0.697**	**0.678**	**0.836**	**-0.764**
15	**-0.369**	**-0.372**	**-0.625**	**0.458**	**0.488**	**-0.495**	**0.664**	**-0.441**	**-0.564**	**-0.590**	**-0.605**	**0.581**
16	**0.579**	**0.561**	**0.799**	**-0.656**	**-0.417**	**0.675**	**-0.564**	**0.539**	**0.555**	**0.488**	**0.825**	**-0.860**
17	-0.034	0.123	0.051	-0.021	0.190	0.002	-0.038	-0.066	0.078	0.009	-0.024	-0.039
18	-0.044	0.139	-0.208	0.283	0.264	**-0.330**	0.084	-0.068	0.044	-0.078	**-0.427**	**0.405**
19	-0.042	0.097	-0.079	0.134	0.230	0.111	0.050	-0.040	-0.201	-0.101	-0.091	0.117
20	0.074	0.007	-0.080	0.005	-0.152	0.081	0.284	0.082	-0.086	0.055	-0.030	-0.087
21	0.019	0.012	-0.215	**0.374**	0.012	-0.142	0.090	-0.036	0.046	0.130	-0.322	0.191
22	-0.018	0.035	0.221	**-0.384**	-0.006	0.260	-0.095	0.106	-0.018	-0.084	**0.365**	-0.169
23	-0.072	-0.099	-0.256	**0.388**	0.091	**-0.334**	0.021	-0.245	0.048	0.122	**-0.439**	0.316
24	0.029	0.095	0.236	**-0.402**	0.087	0.194	-0.209	0.086	0.156	-0.002	0.326	-0.129
25	**-0.537**	**-0.377**	**-0.664**	**0.678**	0.220	**-0.796**	**0.401**	**-0.562**	**-0.339**	**-0.418**	**-0.656**	**0.712**
26	0.164	0.147	0.218	-0.014	-0.143	0.151	-0.136	-0.089	0.103	0.004	0.093	-0.059
27	-0.235	-0.265	-0.112	0.072	-0.147	-0.287	0.178	-0.184	-0.051	-0.166	-0.086	0.146
28	-0.302	**-0.384**	-0.283	0.201	0.214	**-0.374**	0.327	**-0.429**	**-0.425**	**-0.369**	-0.323	0.299
29	-0.238	**-0.366**	-0.227	0.216	0.243	-0.260	0.264	**-0.392**	**-0.415**	**-0.367**	-0.250	0.276
30	**-0.458**	**-0.576**	**-0.418**	0.280	**0.394**	**-0.470**	0.306	**-0.484**	**-0.502**	**-0.415**	**-0.435**	**0.476**
31	**-0.481**	**-0.587**	**-0.426**	0.258	**0.345**	**-0.567**	**0.362**	**-0.501**	**-0.471**	**-0.390**	**-0.447**	**0.473**
32	-0.142	-0.207	-0.048	-0.107	0.155	-0.116	0.129	0.045	-0.141	0.006	-0.104	0.168
33	-0.240	-0.199	-0.281	0.162	-0.023	-0.175	0.062	-0.322	-0.083	0.012	-0.300	0.296
34	-0.145	0.072	-0.101	-0.085	-0.066	0.016	0.053	0.060	0.135	0.068	0.028	-0.059
35	**-0.422**	**-0.527**	**-0.398**	0.219	**0.366**	**-0.531**	**0.337**	**-0.417**	**-0.418**	**-0.372**	**-0.380**	**0.400**
36	0.181	0.026	0.133	-0.249	0.254	0.117	0.087	0.198	0.019	0.122	0.172	-0.202
37	**-0.387**	-0.313	-0.260	0.132	0.002	**-0.461**	0.238	-0.256	-0.295	**-0.512**	-0.232	0.166
38	**-0.389**	**-0.523**	**-0.370**	0.164	**0.369**	**-0.462**	**0.344**	**-0.406**	**-0.432**	**-0.340**	**-0.355**	**0.395**
39	0.190	-0.010	0.031	-0.054	0.251	0.202	0.153	0.121	-0.138	0.004	0.158	-0.149
40	**-0.344**	**-0.454**	-0.322	0.164	0.312	**-0.405**	0.268	-0.299	**-0.338**	-0.292	**-0.332**	**0.356**
41	-0.284	-0.194	-0.098	-0.022	-0.021	-0.317	0.087	0.012	-0.138	-0.266	-0.156	0.101
42	0.138	0.155	0.081	-0.147	-0.250	0.261	-0.091	0.149	0.259	0.291	0.107	-0.123
43	-0.292	**-0.420**	-0.240	0.123	**0.388**	**-0.344**	0.190	**-0.330**	**-0.337**	-0.293	-0.239	0.284
44	0.128	0.115	0.017	0.018	0.153	0.033	0.207	0.272	-0.041	-0.038	0.005	-0.053
45	**-0.427**	**-0.476**	-0.327	0.140	0.263	**-0.503**	0.299	**-0.368**	**-0.359**	**-0.393**	-0.287	0.309
46	**0.469**	**0.370**	**0.499**	**-0.470**	0.088	**0.556**	**-0.373**	**0.633**	**0.403**	**0.453**	**0.386**	**-0.427**
47	0.171	0.275	0.183	0.045	-0.249	0.181	-0.196	0.091	0.164	0.152	0.166	-0.111
48	-0.285	**-0.401**	-0.220	0.126	0.158	**-0.377**	0.143	-0.176	-0.224	-0.325	-0.176	0.189
49	-0.323	-0.252	-0.307	0.289	-0.136	**-0.478**	0.232	-0.196	-0.171	**-0.340**	-0.267	0.263
50	**0.539**	**0.467**	**0.462**	-0.294	0.063	**0.553**	**-0.375**	0.322	0.292	0.275	**0.377**	**-0.428**
51	0.114	0.221	0.171	0.050	-0.281	0.125	-0.211	0.088	0.135	0.119	0.169	-0.098
52	-0.168	-0.278	-0.084	-0.055	0.191	-0.266	0.126	-0.081	-0.154	-0.183	-0.084	0.024
53	**-0.483**	-0.281	-0.281	0.180	0.214	**-0.336**	0.188	**-0.439**	**-0.348**	**-0.382**	**-0.378**	**0.343**
54	-0.223	-0.118	-0.131	0.046	0.180	-0.065	0.126	-0.191	-0.190	-0.200	-0.179	0.154
55	-0.193	0.039	0.012	0.117	0.097	-0.094	-0.029	-0.200	-0.159	-0.268	-0.138	0.102
56	**0.345**	**0.349**	**0.341**	-0.099	-0.174	0.291	-0.269	0.304	0.242	0.160	0.289	-0.288
57	-0.214	0.038	-0.032	0.201	-0.007	-0.121	0.006	-0.228	-0.164	-0.235	-0.140	0.155
58	-0.133	0.034	0.064	-0.006	0.207	-0.044	-0.067	-0.132	-0.126	-0.261	-0.111	0.021
59	-0.176	-0.007	-0.127	0.263	-0.253	-0.129	0.061	-0.161	-0.079	-0.020	-0.059	0.189
60	**-0.371**	-0.143	-0.158	0.153	0.184	-0.223	0.102	**-0.347**	-0.282	**-0.352**	-0.285	0.246
61	**-0.371**	-0.143	-0.158	0.153	0.184	-0.223	0.102	**-0.347**	-0.282	**-0.352**	-0.285	0.246

Numbers in the first column correspond to numbers in the first row; 1, overall aroma intensity; 2, gamey aroma; 3, beef-like aroma; 4, metallic aroma; 5, liver-like aroma; 6, herbaceous aroma; 7, off, sour, sweat-like aroma; 8, sweet-associated aroma; 9, overall flavour intensity; 10, gamey flavour; 11, beef-like flavour; 12, metallic flavour; 13, liver-like flavour; 14, herbaceous flavour; 15, off, sour, sweat-like flavour; 16, sweet associated taste; 17, salty taste; 18, sour taste; 19, initial juiciness; 20, sustained juiciness; 21, tenderness; 22, residue; 23, mealiness; 24, Warner-Bratzler shear force; 25, thaw loss percentage; 26, cooking loss percentage; 27, C6:0; 28, C8:0; 29, C10:0; 30, C12:0; 31, C14:0; 32, C15:0; 33, C16:0; 34, C18:0; 35, C20:0; 36, C22:0; 37, C24:0; 38, C14:1; 39, C15:1; 40, C16:1; 41, C17:1; 42, C18:1n9c; 43, C20:1; 44, C18:2n9c; 45, C18:3n6; 46, C18:3n3; 47, C20:2n6; 48, C20:3n6; 49, C20:3n3; 50, C20:5n3; 51, C22:2n6; 52, C22:6n3; 53, total SFA; 54, total MUFA; 55, total PUFA; 56, PUFA:SFA ratio; 57, n6; 58, n3; 59, n6:n3 ratio; 60, total fatty acids; 61, intramuscular fat content. The non-shaded area indicates Pearson correlation coefficients (*r*); area shaded in grey indicates corresponding P-values for Pearson correlation coefficients (*r*).

Table S1 Continued.

Variable	13	14	15	16	17	18	19	20	21	22	23	24
1	0.247	**<0.001**	**0.027**	**0.000**	0.845	0.797	0.807	0.666	0.912	0.916	0.675	0.868
2	0.346	**<0.001**	**0.025**	**0.000**	0.476	0.418	0.573	0.967	0.943	0.842	0.564	0.580
3	**0.042**	**<0.001**	**<0.001**	**<0.001**	0.766	0.224	0.648	0.644	0.207	0.195	0.131	0.165
4	0.094	**<0.001**	**0.005**	**<0.001**	0.903	0.094	0.436	0.977	**0.025**	**0.021**	**0.019**	**0.015**
5	0.301	**0.047**	**0.003**	**0.011**	0.267	0.119	0.178	0.377	0.947	0.972	0.596	0.613
6	**0.037**	**<0.001**	**0.002**	**<0.001**	0.990	**0.049**	0.518	0.638	0.407	0.126	**0.046**	0.257
7	0.279	**0.000**	**<0.001**	**0.000**	0.827	0.624	0.773	0.093	0.602	0.582	0.903	0.221
8	0.100	**<0.001**	**0.007**	**0.001**	0.702	0.694	0.816	0.634	0.833	0.540	0.149	0.620
9	0.202	**<0.001**	**0.000**	**0.000**	0.651	0.801	0.240	0.619	0.789	0.915	0.781	0.364
10	0.460	**<0.001**	**0.000**	**0.003**	0.957	0.650	0.558	0.750	0.449	0.625	0.479	0.992
11	**0.004**	**<0.001**	**<0.001**	**<0.001**	0.888	**0.009**	0.599	0.863	0.055	**0.029**	**0.007**	0.053
12	0.056	**<0.001**	**0.000**	**<0.001**	0.822	**0.014**	0.497	0.612	0.266	0.323	0.060	0.453
13	**1**	0.076	0.734	**0.025**	1.000	0.174	0.941	0.171	**0.000**	**0.001**	**<0.001**	**0.005**
14	-0.300	**1**	**<0.001**	**<0.001**	0.885	0.063	0.743	0.679	0.587	0.309	0.131	0.370
15	0.059	**-0.611**	**1**	**<0.001**	0.480	**0.041**	0.995	0.950	0.652	0.861	0.838	0.713
16	**-0.374**	**0.799**	**-0.671**	**1**	0.656	**0.006**	0.569	0.766	0.138	0.126	**0.015**	0.266
17	0.000	0.025	-0.122	0.077	**1**	0.620	0.779	0.451	0.969	0.499	0.937	0.109
18	0.232	-0.314	**0.343**	**-0.447**	0.085	**1**	0.799	0.863	0.383	0.141	0.269	0.990
19	-0.013	0.056	-0.001	-0.098	-0.048	-0.044	**1**	0.411	0.712	0.858	0.676	0.808
20	0.233	0.071	0.011	-0.051	-0.130	0.030	0.141	**1**	**<0.001**	**0.000**	0.078	**0.007**
21	**0.569**	-0.094	-0.078	-0.252	0.007	0.150	0.064	**0.610**	**1**	**<0.001**	**<0.001**	**<0.001**
22	**-0.530**	0.174	0.030	0.260	0.116	-0.250	0.031	**-0.567**	**-0.916**	**1**	**<0.001**	**<0.001**
23	**0.692**	-0.256	-0.035	**-0.401**	0.014	0.189	-0.072	0.298	**0.838**	**-0.829**	**1**	**0.000**
24	**-0.460**	0.154	-0.063	0.190	0.272	0.002	-0.042	**-0.445**	**-0.745**	**0.779**	**-0.577**	**1**
25	**0.348**	**-0.658**	**0.400**	**-0.582**	0.041	**0.487**	-0.028	-0.289	0.131	-0.196	0.303	-0.142
26	-0.004	0.178	-0.151	0.156	0.096	-0.161	-0.124	**-0.534**	**-0.354**	**0.374**	-0.176	0.312
27	-0.115	-0.181	0.097	-0.114	0.078	**0.365**	-0.317	0.192	0.050	-0.190	-0.058	-0.109
28	0.251	**-0.388**	**0.382**	**-0.438**	-0.050	-0.140	-0.200	-0.204	-0.094	0.063	0.159	-0.145
29	0.177	-0.317	0.277	**-0.350**	-0.002	-0.224	-0.085	-0.283	-0.176	0.144	0.065	-0.038
30	0.210	**-0.522**	**0.412**	**-0.547**	0.008	-0.117	-0.083	-0.276	-0.101	0.044	0.214	-0.020
31	0.210	**-0.567**	**0.418**	**-0.565**	-0.019	0.038	-0.189	-0.119	-0.030	-0.100	0.245	-0.107
32	0.082	-0.058	0.247	-0.169	-0.048	-0.015	-0.159	-0.191	-0.173	0.269	-0.065	0.205
33	**0.412**	-0.175	0.061	-0.310	0.147	-0.064	-0.001	-0.138	0.234	-0.147	**0.427**	0.023
34	0.160	0.030	-0.094	0.012	0.246	-0.048	0.098	0.110	0.176	-0.045	0.178	-0.037
35	0.208	**-0.527**	**0.448**	**-0.551**	-0.092	-0.016	-0.191	-0.121	-0.051	-0.032	0.247	-0.122
36	-0.093	0.118	-0.002	0.025	-0.013	-0.108	-0.122	0.160	0.009	-0.037	0.021	-0.085
37	-0.045	**-0.424**	**0.329**	-0.305	-0.226	0.069	-0.269	0.008	-0.153	0.051	-0.041	-0.097
38	0.192	**-0.477**	**0.434**	**-0.516**	-0.061	-0.047	-0.185	-0.127	-0.068	-0.022	0.215	-0.086
39	-0.009	0.016	0.091	0.034	-0.104	-0.301	0.022	0.125	-0.073	0.091	-0.051	-0.156
40	0.285	**-0.446**	**0.366**	**-0.544**	-0.097	0.019	-0.209	0.050	0.081	-0.111	0.314	-0.167
41	0.049	-0.256	0.304	-0.172	-0.167	0.170	**-0.340**	0.005	-0.080	0.110	-0.064	0.003
42	**0.338**	0.222	-0.123	0.045	0.123	-0.024	-0.061	0.225	0.296	-0.125	0.283	-0.004
43	0.105	**-0.381**	0.323	**-0.382**	-0.126	-0.176	-0.125	-0.177	-0.132	0.075	0.189	-0.048
44	-0.213	0.046	0.289	-0.010	-0.288	0.231	-0.045	0.073	0.007	-0.041	-0.177	-0.266
45	0.098	**-0.472**	**0.383**	**-0.470**	-0.087	0.002	-0.263	-0.109	-0.109	0.029	0.154	-0.078
46	-0.144	**0.477**	-0.252	0.260	-0.037	0.032	0.023	0.228	0.145	-0.106	-0.031	0.013
47	-0.280	0.215	-0.302	0.310	0.125	0.126	0.246	0.006	-0.094	0.076	-0.323	0.163
48	0.018	**-0.400**	0.296	**-0.388**	-0.299	0.006	-0.249	0.048	-0.038	-0.081	0.130	-0.204
49	0.046	**-0.378**	0.211	-0.225	-0.223	0.324	-0.177	0.064	0.034	-0.148	-0.014	-0.263
50	-0.306	**0.525**	-0.325	**0.599**	0.004	-0.292	0.203	-0.170	-0.189	0.192	-0.236	0.258
51	-0.278	0.170	-0.279	0.276	0.051	0.138	0.230	0.028	-0.110	0.070	**-0.338**	0.152
52	0.063	-0.268	0.208	-0.274	-0.125	0.012	-0.262	0.068	0.010	-0.102	0.174	-0.199
53	0.254	**-0.340**	0.325	-0.303	0.277	-0.023	0.102	-0.321	-0.188	0.255	0.023	0.256
54	0.288	-0.154	0.280	-0.184	0.202	-0.072	0.056	-0.100	-0.088	0.219	0.052	0.186
55	-0.095	-0.079	0.101	0.082	0.221	0.059	0.252	-0.316	**-0.342**	**0.349**	**-0.333**	**0.331**
56	**-0.409**	0.303	-0.254	**0.419**	-0.119	0.130	0.133	0.077	-0.124	0.040	**-0.379**	0.053
57	-0.132	-0.097	0.064	0.044	0.234	0.134	0.286	-0.256	-0.308	0.315	**-0.370**	0.304
58	-0.034	-0.043	0.129	0.114	0.166	-0.043	0.167	**-0.333**	-0.324	**0.330**	-0.230	0.307
59	-0.153	-0.090	-0.056	-0.062	0.148	0.214	0.243	0.028	-0.072	0.083	-0.256	0.101
60	0.132	-0.234	0.259	-0.144	0.277	0.004	0.179	-0.326	-0.270	**0.330**	-0.136	0.316
61	0.132	-0.234	0.259	-0.144	0.277	0.004	0.179	-0.326	-0.270	**0.330**	-0.136	0.316

Numbers in the first column correspond to numbers in the first row; 1, overall aroma intensity; 2, gamey aroma; 3, beef-like aroma; 4, metallic aroma; 5, liver-like aroma; 6, herbaceous aroma; 7, off, sour, sweat-like aroma; 8, sweet-associated aroma; 9, overall flavour intensity; 10, gamey flavour; 11, beef-like flavour; 12, metallic flavour; 13, liver-like flavour; 14, herbaceous flavour; 15, off, sour, sweat-like flavour; 16, sweet associated taste; 17, salty taste; 18, sour taste; 19, initial juiciness; 20, sustained juiciness; 21, tenderness; 22, residue; 23, mealiness; 24, Warner-Bratzler shear force; 25, thaw loss percentage; 26, cooking loss percentage; 27, C6:0; 28, C8:0; 29, C10:0; 30, C12:0; 31, C14:0; 32, C15:0; 33, C16:0; 34, C18:0; 35, C20:0; 36, C22:0; 37, C24:0; 38, C14:1; 39, C15:1; 40, C16:1; 41, C17:1; 42, C18:1n9c; 43, C20:1; 44, C18:2n9c; 45, C18:3n6; 46, C18:3n3; 47, C20:2n6; 48, C20:3n6; 49, C20:3n3; 50, C20:5n3; 51, C22:2n6; 52, C22:6n3; 53, total SFA; 54, total MUFA; 55, total PUFA; 56, PUFA:SFA ratio; 57, n6; 58, n3; 59, n6:n3 ratio; 60, total fatty acids; 61, intramuscular fat content. The non-shaded area indicates Pearson correlation coefficients (*r*); area shaded in grey indicates corresponding P-values for Pearson correlation coefficients (*r*).

Table S1 Continued.

Variable	25	26	27	28	29	30	31	32	33	34	35	36
1	**0.001**	0.339	0.167	0.073	0.162	**0.005**	**0.003**	0.409	0.158	0.397	**0.010**	0.292
2	**0.024**	0.392	0.119	**0.021**	**0.028**	**0.000**	**0.000**	0.226	0.245	0.677	**0.001**	0.881
3	**<0.001**	0.202	0.516	0.094	0.183	**0.011**	**0.009**	0.783	0.097	0.560	**0.016**	0.438
4	**<0.001**	0.938	0.677	0.241	0.205	0.098	0.129	0.534	0.346	0.621	0.199	0.144
5	0.197	0.404	0.394	0.211	0.153	**0.017**	**0.040**	0.368	0.893	0.703	**0.028**	0.135
6	**<0.001**	0.379	0.089	**0.024**	0.125	**0.004**	**0.000**	0.499	0.308	0.928	**0.001**	0.497
7	**0.015**	0.429	0.298	0.052	0.119	0.069	**0.030**	0.453	0.720	0.759	**0.045**	0.614
8	**0.000**	0.605	0.282	**0.009**	**0.018**	**0.003**	**0.002**	0.795	0.055	0.730	**0.011**	0.248
9	**0.043**	0.551	0.766	**0.010**	**0.012**	**0.002**	**0.004**	0.413	0.629	0.432	**0.011**	0.914
10	**0.011**	0.981	0.333	**0.027**	**0.028**	**0.012**	**0.019**	0.974	0.946	0.692	**0.026**	0.478
11	**<0.001**	0.589	0.618	0.055	0.142	**0.008**	**0.006**	0.547	0.075	0.870	**0.022**	0.316
12	**<0.001**	0.735	0.394	0.076	0.103	**0.003**	**0.004**	0.328	0.080	0.731	**0.016**	0.237
13	**0.038**	0.983	0.502	0.140	0.301	0.219	0.219	0.636	**0.012**	0.351	0.224	0.589
14	**<0.001**	0.300	0.291	**0.019**	0.060	**0.001**	**0.000**	0.735	0.309	0.860	**0.001**	0.492
15	**0.016**	0.380	0.573	**0.022**	0.102	**0.013**	**0.011**	0.147	0.725	0.586	**0.006**	0.990
16	**0.000**	0.363	0.508	**0.008**	**0.036**	**0.001**	**0.000**	0.324	0.066	0.943	**0.000**	0.885
17	0.814	0.577	0.652	0.774	0.991	0.964	0.912	0.781	0.392	0.148	0.593	0.939
18	**0.003**	0.349	**0.028**	0.415	0.190	0.497	0.825	0.929	0.710	0.780	0.925	0.530
19	0.870	0.472	0.059	0.243	0.623	0.631	0.271	0.354	0.995	0.570	0.265	0.480
20	0.088	**0.001**	0.261	0.233	0.094	0.104	0.489	0.264	0.423	0.523	0.482	0.351
21	0.446	**0.034**	0.770	0.587	0.304	0.558	0.861	0.312	0.170	0.305	0.769	0.957
22	0.253	**0.024**	0.267	0.717	0.401	0.801	0.563	0.113	0.392	0.795	0.853	0.828
23	0.072	0.304	0.736	0.353	0.708	0.211	0.150	0.706	**0.009**	0.298	0.146	0.903
24	0.407	0.064	0.527	0.400	0.825	0.908	0.533	0.230	0.896	0.829	0.480	0.621
25	**1**	0.587	0.402	0.365	0.537	0.109	0.110	0.630	0.139	0.976	0.220	0.050
26	0.094	**1**	0.100	0.100	**0.004**	0.362	0.516	0.566	0.302	0.094	0.408	0.055
27	0.144	-0.279	**1**	0.921	0.782	0.895	0.094	0.706	**0.014**	0.134	0.560	0.548
28	0.156	0.279	0.017	**1**	**<0.001**	**<0.001**	**<0.001**	**0.030**	0.590	0.619	**<0.001**	**0.050**
29	0.106	**0.467**	-0.048	**0.939**	**1**	**<0.001**	**<0.001**	0.055	0.739	0.208	**<0.001**	0.136
30	0.272	0.156	0.023	**0.878**	**0.867**	**1**	**<0.001**	**0.006**	0.253	0.489	**<0.001**	0.067
31	0.271	-0.112	0.283	**0.791**	**0.707**	**0.906**	**1**	**0.014**	0.729	0.440	**<0.001**	**0.004**
32	0.083	0.099	0.065	**0.361**	0.323	**0.447**	**0.406**	**1**	0.276	0.282	**0.015**	**0.017**
33	0.252	0.177	**-0.404**	0.093	0.058	0.195	0.060	-0.187	**1**	**0.025**	0.648	**0.040**
34	0.005	-0.283	-0.254	-0.086	-0.215	-0.119	-0.133	-0.184	**0.373**	**1**	0.949	0.570
35	0.209	-0.142	0.101	**0.843**	**0.703**	**0.875**	**0.930**	**0.401**	0.079	0.011	**1**	**0.001**
36	-0.329	-0.323	0.103	**0.330**	0.253	0.308	**0.474**	**0.394**	**-0.344**	0.098	**0.527**	**1**
37	0.191	-0.123	0.313	**0.508**	**0.337**	**0.342**	**0.427**	0.092	-0.269	0.030	**0.539**	0.081
38	0.176	-0.077	0.139	**0.856**	**0.759**	**0.928**	**0.965**	**0.471**	0.055	-0.049	**0.971**	**0.549**
39	**-0.353**	-0.117	-0.311	**0.431**	**0.410**	0.302	0.276	0.029	-0.218	0.057	**0.428**	**0.564**
40	0.093	-0.189	0.048	**0.808**	**0.647**	**0.788**	**0.844**	0.313	0.178	0.102	**0.928**	**0.513**
41	0.176	-0.131	0.265	**0.341**	0.149	0.237	0.267	**0.562**	-0.280	-0.015	**0.372**	0.074
42	-0.179	-0.007	**-0.385**	-0.154	-0.246	-0.266	**-0.353**	-0.273	**0.664**	**0.633**	-0.218	-0.207
43	0.078	-0.044	-0.065	**0.810**	**0.720**	**0.869**	**0.861**	**0.407**	0.056	-0.020	**0.939**	**0.549**
44	0.029	-0.292	0.258	-0.126	-0.216	-0.199	-0.064	0.269	**-0.550**	-0.279	-0.010	0.269
45	0.182	-0.140	0.226	**0.834**	**0.669**	**0.806**	**0.876**	**0.372**	-0.019	0.087	**0.958**	**0.520**
46	**-0.559**	**-0.330**	-0.120	-0.327	**-0.350**	-0.303	-0.215	0.037	-0.088	-0.003	-0.164	**0.414**
47	0.013	0.177	0.162	**-0.660**	**-0.469**	**-0.618**	**-0.606**	**-0.352**	-0.273	**-0.381**	**-0.786**	**-0.522**
48	0.015	-0.270	0.229	**0.653**	**0.487**	**0.559**	**0.667**	0.142	-0.191	-0.011	**0.800**	**0.435**
49	**0.422**	-0.140	**0.561**	0.076	-0.014	-0.028	0.100	0.068	**-0.479**	-0.161	0.086	-0.186
50	-0.291	0.296	**-0.356**	**-0.499**	-0.292	**-0.360**	**-0.487**	-0.053	-0.086	-0.297	**-0.563**	-0.145
51	0.006	0.138	0.208	**-0.605**	**-0.429**	**-0.579**	**-0.554**	**-0.357**	-0.317	**-0.433**	**-0.722**	**-0.530**
52	-0.082	**-0.352**	0.236	**0.622**	**0.432**	**0.535**	**0.675**	0.267	-0.229	0.097	**0.810**	**0.650**
53	0.256	0.283	-0.081	**0.509**	**0.512**	**0.579**	**0.405**	0.318	**0.340**	0.160	**0.399**	-0.148
54	-0.021	0.164	-0.282	**0.457**	**0.410**	**0.407**	0.229	0.199	**0.350**	0.315	**0.338**	-0.038
55	0.158	**0.363**	0.096	-0.016	0.129	0.054	-0.081	0.156	-0.211	-0.323	-0.191	**-0.415**
56	-0.149	0.006	0.275	**-0.640**	**-0.500**	**-0.640**	**-0.562**	-0.227	**-0.615**	**-0.563**	**-0.683**	-0.304
57	0.201	**0.360**	0.173	-0.125	0.038	-0.080	-0.182	0.005	-0.222	**-0.373**	**-0.330**	**-0.522**
58	0.078	0.304	-0.015	0.120	0.217	0.209	0.057	0.315	-0.161	-0.204	0.012	-0.212
59	0.185	0.097	0.228	**-0.350**	-0.244	**-0.360**	**-0.333**	**-0.349**	-0.104	-0.228	**-0.472**	**-0.504**
60	0.198	**0.341**	-0.045	0.326	**0.388**	**0.385**	0.204	0.267	0.131	-0.017	0.168	-0.276
61	0.198	**0.341**	-0.045	0.326	**0.388**	**0.385**	0.204	0.267	0.131	-0.017	0.168	-0.276

Numbers in the first column correspond to numbers in the first row; 1, overall aroma intensity; 2, gamey aroma; 3, beef-like aroma; 4, metallic aroma; 5, liver-like aroma; 6, herbaceous aroma; 7, off, sour, sweat-like aroma; 8, sweet-associated aroma; 9, overall flavour intensity; 10, gamey flavour; 11, beef-like flavour; 12, metallic flavour; 13, liver-like flavour; 14, herbaceous flavour; 15, off, sour, sweat-like flavour; 16, sweet associated taste; 17, salty taste; 18, sour taste; 19, initial juiciness; 20, sustained juiciness; 21, tenderness; 22, residue; 23, mealiness; 24, Warner-Bratzler shear force; 25, thaw loss percentage; 26, cooking loss percentage; 27, C6:0; 28, C8:0; 29, C10:0; 30, C12:0; 31, C14:0; 32, C15:0; 33, C16:0; 34, C18:0; 35, C20:0; 36, C22:0; 37, C24:0; 38, C14:1; 39, C15:1; 40, C16:1; 41, C17:1; 42, C18:1n9c; 43, C20:1; 44, C18:2n9c; 45, C18:3n6; 46, C18:3n3; 47, C20:2n6; 48, C20:3n6; 49, C20:3n3; 50, C20:5n3; 51, C22:2n6; 52, C22:6n3; 53, total SFA; 54, total MUFA; 55, total PUFA; 56, PUFA:SFA ratio; 57, n6; 58, n3; 59, n6:n3 ratio; 60, total fatty acids; 61, intramuscular fat content. The non-shaded area indicates Pearson correlation coefficients (*r*); area shaded in grey indicates corresponding P-values for Pearson correlation coefficients (*r*).

Table S1 Continued.

Variable	37	38	39	40	41	42	43	44	45	46	47	48
1	**0.020**	**0.019**	0.268	**0.040**	0.093	0.424	0.084	0.455	**0.009**	**0.004**	0.318	0.092
2	0.063	**0.001**	0.952	**0.005**	0.257	0.366	**0.011**	0.503	**0.003**	**0.027**	0.104	**0.015**
3	0.126	**0.026**	0.858	0.056	0.571	0.641	0.158	0.923	0.051	**0.002**	0.287	0.198
4	0.442	0.340	0.753	0.340	0.897	0.392	0.474	0.918	0.415	**0.004**	0.796	0.464
5	0.991	**0.027**	0.140	0.064	0.904	0.141	**0.019**	0.373	0.121	0.610	0.143	0.356
6	**0.005**	**0.005**	0.237	**0.014**	0.059	0.125	**0.040**	0.848	**0.002**	**0.000**	0.292	**0.024**
7	0.163	**0.040**	0.373	0.114	0.615	0.598	0.268	0.225	0.076	**0.025**	0.252	0.404
8	0.131	**0.014**	0.482	0.077	0.943	0.386	**0.049**	0.108	**0.027**	**<0.001**	0.598	0.304
9	0.081	**0.009**	0.422	**0.044**	0.422	0.127	**0.044**	0.814	**0.032**	**0.015**	0.340	0.188
10	**0.001**	**0.042**	0.981	0.084	0.117	0.085	0.083	0.824	**0.018**	**0.006**	0.377	0.053
11	0.173	**0.033**	0.358	**0.048**	0.363	0.534	0.161	0.977	0.090	**0.020**	0.333	0.304
12	0.333	**0.017**	0.386	**0.033**	0.556	0.475	0.093	0.758	0.067	**0.009**	0.520	0.269
13	0.792	0.263	0.957	0.091	0.776	**0.044**	0.541	0.211	0.569	0.402	0.098	0.918
14	**0.010**	**0.003**	0.926	**0.006**	0.132	0.194	**0.022**	0.790	**0.004**	**0.003**	0.209	**0.016**
15	**0.050**	**0.008**	0.598	**0.028**	0.071	0.474	0.055	0.087	**0.021**	0.137	0.074	0.080
16	0.071	**0.001**	0.844	**0.001**	0.317	0.795	**0.022**	0.955	**0.004**	0.125	0.065	**0.019**
17	0.184	0.725	0.547	0.572	0.329	0.476	0.464	0.088	0.613	0.830	0.469	0.076
18	0.688	0.787	0.074	0.914	0.323	0.890	0.305	0.176	0.989	0.854	0.465	0.971
19	0.113	0.280	0.900	0.220	**0.043**	0.722	0.467	0.794	0.121	0.895	0.149	0.143
20	0.961	0.461	0.469	0.771	0.976	0.187	0.302	0.673	0.528	0.181	0.970	0.781
21	0.374	0.692	0.673	0.637	0.642	0.079	0.444	0.966	0.526	0.398	0.586	0.825
22	0.770	0.900	0.599	0.518	0.522	0.467	0.662	0.812	0.866	0.538	0.661	0.639
23	0.813	0.209	0.767	0.063	0.709	0.095	0.269	0.301	0.370	0.860	0.055	0.450
24	0.572	0.617	0.362	0.329	0.988	0.983	0.779	0.116	0.651	0.939	0.343	0.233
25	0.264	0.303	**0.035**	0.590	0.305	0.297	0.649	0.868	0.288	**0.000**	0.940	0.932
26	0.473	0.655	0.495	0.269	0.447	0.968	0.800	0.084	0.416	**0.049**	0.303	0.112
27	0.063	0.419	0.065	0.782	0.118	**0.020**	0.708	0.129	0.185	0.486	0.346	0.179
28	**0.002**	**<0.001**	**0.009**	**<0.001**	**0.042**	0.369	**<0.001**	0.463	**<0.001**	0.052	**<0.001**	**<0.001**
29	**0.045**	**<0.001**	**0.013**	**<0.001**	0.385	0.149	**<0.001**	0.205	**<0.001**	**0.036**	**0.004**	**0.003**
30	**0.041**	**<0.001**	0.074	**<0.001**	0.165	0.116	**<0.001**	0.245	**<0.001**	0.072	**<0.001**	**0.000**
31	**0.009**	**<0.001**	0.103	**<0.001**	0.115	**0.035**	**<0.001**	0.711	**<0.001**	0.207	**<0.001**	**<0.001**
32	0.594	**0.004**	0.869	0.063	**0.000**	0.107	**0.014**	0.112	**0.025**	0.830	**0.035**	0.407
33	0.112	0.748	0.202	0.300	0.098	**<0.001**	0.744	**0.001**	0.913	0.611	0.107	0.264
34	0.861	0.778	0.741	0.555	0.933	**<0.001**	0.906	0.100	0.612	0.987	**0.022**	0.949
35	**0.001**	**<0.001**	**0.009**	**<0.001**	**0.025**	0.201	**<0.001**	0.953	**<0.001**	0.338	**<0.001**	**<0.001**
36	0.637	**0.001**	**0.000**	**0.001**	0.669	0.227	**0.001**	0.113	**0.001**	**0.012**	**0.001**	**0.008**
37	**1**	**0.006**	0.830	**0.004**	**<0.001**	0.168	**0.016**	0.395	**<0.001**	**0.036**	**0.012**	**<0.001**
38	**0.453**	**1**	**0.014**	**<0.001**	0.055	0.108	**<0.001**	0.840	**<0.001**	0.331	**<0.001**	**<0.001**
39	0.037	**0.406**	**1**	**0.003**	0.817	0.972	**0.001**	0.685	**0.024**	0.354	**0.006**	**0.021**
40	**0.470**	**0.889**	**0.482**	**1**	**0.024**	0.895	**<0.001**	0.472	**<0.001**	0.964	**<0.001**	**<0.001**
41	**0.656**	0.322	-0.040	**0.376**	**1**	0.418	0.120	0.124	**0.003**	0.674	**0.019**	**0.002**
42	-0.235	-0.273	0.006	0.023	-0.139	**1**	0.161	**0.014**	0.215	0.257	0.115	0.232
43	**0.399**	**0.928**	**0.532**	**0.871**	0.264	-0.239	**1**	0.684	**<0.001**	0.367	**<0.001**	**<0.001**
44	0.146	-0.035	0.070	-0.124	0.261	**-0.408**	-0.070	**1**	0.943	0.170	0.999	0.651
45	**0.673**	**0.922**	**0.375**	**0.900**	**0.489**	-0.212	**0.889**	0.012	**1**	0.207	**<0.001**	**<0.001**
46	**-0.350**	-0.167	0.159	-0.008	-0.073	0.194	-0.155	0.234	-0.216	**1**	0.471	0.813
47	**-0.416**	**-0.741**	**-0.448**	**-0.819**	**-0.389**	-0.268	**-0.750**	0.000	**-0.767**	-0.124	**1**	**<0.001**
48	**0.798**	**0.716**	**0.383**	**0.776**	**0.500**	-0.204	**0.697**	0.078	**0.844**	-0.041	**-0.653**	**1**
49	**0.709**	0.044	-0.322	-0.035	**0.545**	**-0.431**	-0.100	**0.390**	0.205	**-0.401**	0.100	**0.375**
50	**-0.632**	**-0.480**	-0.107	**-0.637**	**-0.486**	-0.138	-0.321	-0.003	**-0.649**	0.184	**0.459**	**-0.663**
51	-0.314	**-0.691**	**-0.428**	**-0.752**	-0.296	-0.317	**-0.708**	0.018	**-0.692**	-0.129	**0.979**	**-0.536**
52	**0.676**	**0.746**	**0.446**	**0.783**	**0.495**	-0.165	**0.708**	0.206	**0.852**	0.154	**-0.761**	**0.903**
53	0.154	**0.421**	0.001	**0.342**	0.270	0.081	**0.379**	**-0.434**	**0.370**	**-0.338**	**-0.340**	0.108
54	0.073	0.321	**0.349**	**0.415**	0.252	**0.423**	**0.351**	**-0.420**	0.310	-0.100	**-0.486**	0.143
55	-0.049	-0.142	-0.230	-0.329	0.114	**-0.371**	-0.154	-0.130	-0.200	-0.315	**0.411**	-0.286
56	-0.202	**-0.657**	-0.313	**-0.752**	-0.160	**-0.477**	**-0.645**	**0.378**	**-0.650**	0.106	**0.844**	**-0.401**
57	-0.119	-0.284	-0.298	**-0.442**	-0.005	**-0.380**	-0.317	-0.125	-0.321	**-0.385**	**0.639**	**-0.383**
58	0.045	0.058	-0.106	-0.132	0.240	-0.295	0.074	-0.114	-0.016	-0.174	0.059	-0.118
59	-0.211	**-0.468**	-0.315	**-0.473**	-0.261	-0.173	**-0.520**	-0.067	**-0.428**	**-0.346**	**0.811**	**-0.397**
60	0.066	0.200	-0.046	0.089	0.232	-0.060	0.178	**-0.352**	0.145	**-0.335**	-0.061	-0.056
61	0.066	0.200	-0.046	0.089	0.232	-0.060	0.178	**-0.352**	0.145	**-0.335**	-0.061	-0.056

Numbers in the first column correspond to numbers in the first row; 1, overall aroma intensity; 2, gamey aroma; 3, beef-like aroma; 4, metallic aroma; 5, liver-like aroma; 6, herbaceous aroma; 7, off, sour, sweat-like aroma; 8, sweet-associated aroma; 9, overall flavour intensity; 10, gamey flavour; 11, beef-like flavour; 12, metallic flavour; 13, liver-like flavour; 14, herbaceous flavour; 15, off, sour, sweat-like flavour; 16, sweet associated taste; 17, salty taste; 18, sour taste; 19, initial juiciness; 20, sustained juiciness; 21, tenderness; 22, residue; 23, mealiness; 24, Warner-Bratzler shear force; 25, thaw loss percentage; 26, cooking loss percentage; 27, C6:0; 28, C8:0; 29, C10:0; 30, C12:0; 31, C14:0; 32, C15:0; 33, C16:0; 34, C18:0; 35, C20:0; 36, C22:0; 37, C24:0; 38, C14:1; 39, C15:1; 40, C16:1; 41, C17:1; 42, C18:1n9c; 43, C20:1; 44, C18:2n9c; 45, C18:3n6; 46, C18:3n3; 47, C20:2n6; 48, C20:3n6; 49, C20:3n3; 50, C20:5n3; 51, C22:2n6; 52, C22:6n3; 53, total SFA; 54, total MUFA; 55, total PUFA; 56, PUFA:SFA ratio; 57, n6; 58, n3; 59, n6:n3 ratio; 60, total fatty acids; 61, intramuscular fat content. The non-shaded area indicates Pearson correlation coefficients (*r*); area shaded in grey indicates corresponding P-values for Pearson correlation coefficients (*r*).

Table S1 Continued.

Variable	49	50	51	52	53	54	55	56	57	58	59	60	61
1	0.054	**0.001**	0.507	0.328	**0.003**	0.191	0.260	**0.040**	0.210	0.440	0.306	**0.026**	**0.026**
2	0.138	**0.004**	0.196	0.101	0.096	0.492	0.820	**0.037**	0.828	0.842	0.968	0.407	0.407
3	0.069	**0.005**	0.319	0.627	0.097	0.445	0.945	**0.042**	0.851	0.709	0.461	0.358	0.358
4	0.088	0.082	0.772	0.749	0.294	0.788	0.495	0.564	0.239	0.972	0.122	0.373	0.373
5	0.430	0.716	0.096	0.263	0.211	0.294	0.575	0.310	0.968	0.225	0.137	0.284	0.284
6	**0.003**	**0.000**	0.468	0.116	**0.045**	0.709	0.586	0.085	0.483	0.797	0.454	0.192	0.192
7	0.174	**0.024**	0.217	0.466	0.273	0.463	0.866	0.112	0.972	0.697	0.724	0.554	0.554
8	0.252	0.055	0.610	0.638	**0.007**	0.264	0.241	0.072	0.181	0.444	0.348	**0.038**	**0.038**
9	0.319	0.084	0.431	0.369	**0.037**	0.266	0.353	0.156	0.339	0.464	0.646	0.095	0.095
10	**0.042**	0.104	0.489	0.286	**0.021**	0.243	0.114	0.350	0.167	0.125	0.909	**0.035**	**0.035**
11	0.115	**0.024**	0.325	0.625	**0.023**	0.297	0.423	0.087	0.414	0.520	0.732	0.092	0.092
12	0.122	**0.009**	0.570	0.891	**0.041**	0.370	0.552	0.089	0.368	0.905	0.271	0.148	0.148
13	0.789	0.070	0.101	0.717	0.136	0.089	0.580	**0.013**	0.444	0.844	0.374	0.443	0.443
14	**0.023**	**0.001**	0.322	0.114	**0.042**	0.369	0.646	0.072	0.572	0.802	0.600	0.170	0.170
15	0.216	0.053	0.100	0.224	0.053	0.098	0.557	0.135	0.710	0.454	0.746	0.128	0.128
16	0.188	**0.000**	0.103	0.106	0.073	0.284	0.634	**0.011**	0.797	0.507	0.720	0.401	0.401
17	0.190	0.982	0.768	0.469	0.102	0.239	0.196	0.491	0.169	0.335	0.388	0.102	0.102
18	0.054	0.084	0.422	0.943	0.894	0.678	0.734	0.449	0.437	0.801	0.210	0.983	0.983
19	0.302	0.234	0.176	0.122	0.554	0.747	0.138	0.440	0.091	0.331	0.153	0.295	0.295
20	0.710	0.322	0.873	0.693	0.056	0.561	0.061	0.654	0.131	**0.047**	0.870	0.052	0.052
21	0.846	0.271	0.523	0.956	0.273	0.611	**0.041**	0.470	0.067	0.054	0.675	0.111	0.111
22	0.390	0.261	0.685	0.555	0.134	0.198	**0.037**	0.817	0.062	**0.049**	0.629	**0.049**	**0.049**
23	0.936	0.166	**0.044**	0.309	0.893	0.762	**0.047**	**0.023**	**0.027**	0.178	0.131	0.429	0.429
24	0.121	0.128	0.378	0.243	0.132	0.277	**0.049**	0.760	0.072	0.069	0.558	0.060	0.060
25	**0.010**	0.085	0.970	0.635	0.132	0.902	0.357	0.384	0.239	0.652	0.280	0.246	0.246
26	0.416	0.079	0.420	**0.035**	0.095	0.338	**0.029**	0.972	**0.031**	0.071	0.574	**0.042**	**0.042**
27	**0.000**	**0.033**	0.222	0.166	0.640	0.095	0.577	0.104	0.312	0.930	0.181	0.796	0.796
28	0.658	**0.002**	**<0.001**	**<0.001**	**0.002**	**0.005**	0.925	**<0.001**	0.467	0.485	**0.037**	0.052	0.052
29	0.936	0.084	**0.009**	**0.008**	**0.001**	**0.013**	0.455	**0.002**	0.824	0.204	0.151	**0.019**	**0.019**
30	0.870	**0.031**	**0.000**	**0.001**	**0.000**	**0.014**	0.755	**<0.001**	0.643	0.222	**0.031**	**0.020**	**0.020**
31	0.563	**0.003**	**0.000**	**<0.001**	**0.014**	0.180	0.637	**0.000**	0.287	0.742	**0.047**	0.233	0.233
32	0.695	0.757	**0.033**	0.115	0.059	0.245	0.364	0.184	0.979	0.061	**0.037**	0.116	0.116
33	**0.003**	0.616	0.060	0.179	**0.043**	**0.037**	0.216	**<0.001**	0.193	0.349	0.545	0.445	0.445
34	0.348	0.079	**0.008**	0.575	0.351	0.061	0.055	**0.000**	**0.025**	0.232	0.181	0.923	0.923
35	0.616	**0.000**	**<0.001**	**<0.001**	**0.016**	**0.044**	0.263	**<0.001**	**0.049**	0.945	**0.004**	0.327	0.327
36	0.278	0.398	**0.001**	**<0.001**	0.389	0.826	**0.012**	0.072	**0.001**	0.215	**0.002**	0.103	0.103
37	**<0.001**	**<0.001**	0.062	**<0.001**	0.370	0.673	0.777	0.237	0.491	0.794	0.217	0.700	0.700
38	0.797	**0.003**	**<0.001**	**<0.001**	**0.011**	0.056	0.410	**<0.001**	0.093	0.738	**0.004**	0.243	0.243
39	0.056	0.535	**0.009**	**0.006**	0.996	**0.037**	0.177	0.063	0.077	0.536	0.062	0.788	0.788
40	0.838	**<0.001**	**<0.001**	**<0.001**	**0.041**	**0.012**	0.050	**<0.001**	**0.007**	0.442	**0.004**	0.607	0.607
41	**0.001**	**0.003**	0.079	**0.002**	0.112	0.138	0.508	0.351	0.978	0.159	0.124	0.172	0.172
42	**0.009**	0.423	0.060	0.336	0.638	**0.010**	**0.026**	**0.003**	**0.022**	0.081	0.313	0.728	0.728
43	0.563	0.056	**<0.001**	**<0.001**	**0.023**	**0.036**	0.371	**<0.001**	0.060	0.669	**0.001**	0.299	0.299
44	**0.019**	0.985	0.919	0.229	**0.008**	**0.011**	0.448	**0.023**	0.466	0.508	0.700	**0.036**	**0.036**
45	0.231	**<0.001**	**<0.001**	**<0.001**	**0.027**	0.066	0.243	**<0.001**	0.056	0.925	**0.009**	0.400	0.400
46	**0.015**	0.282	0.452	0.370	**0.044**	0.560	0.061	0.537	**0.020**	0.311	**0.039**	**0.046**	**0.046**
47	0.563	**0.005**	**<0.001**	**<0.001**	**0.043**	**0.003**	**0.013**	**<0.001**	**<0.001**	0.734	**<0.001**	0.723	0.723
48	**0.024**	**<0.001**	**0.001**	**<0.001**	0.529	0.406	0.090	**0.015**	**0.021**	0.491	**0.017**	0.746	0.746
49	**1**	**0.007**	0.309	0.110	0.533	0.065	0.456	0.077	0.346	0.707	0.393	0.782	0.782
50	**-0.443**	**1**	**0.031**	**<0.001**	0.360	0.261	0.052	**0.002**	0.121	**0.038**	0.856	0.809	0.809
51	0.174	**0.359**	**1**	**<0.001**	**0.040**	**0.002**	**0.015**	**<0.001**	**<0.001**	0.833	**<0.001**	0.694	0.694
52	0.271	**-0.624**	**-0.678**	**1**	0.641	0.394	**0.029**	**0.004**	**0.002**	0.473	**0.000**	0.540	0.540
53	-0.107	-0.157	**-0.344**	0.080	**1**	**<0.001**	**<0.001**	**0.001**	**0.006**	**<0.001**	0.264	**<0.001**	**<0.001**
54	-0.311	-0.192	**-0.490**	0.146	**0.845**	**1**	**0.028**	**<0.001**	0.264	**0.001**	0.062	**<0.001**	**<0.001**
55	0.128	0.326	**0.403**	**-0.364**	**0.618**	**0.366**	**1**	**0.043**	**<0.001**	**<0.001**	0.112	**<0.001**	**<0.001**
56	0.298	**0.495**	**0.854**	**-0.465**	**-0.520**	**-0.612**	**0.340**	**1**	**0.003**	0.520	**0.001**	0.227	0.227
57	0.162	0.263	**0.644**	**-0.508**	**0.446**	0.191	**0.937**	**0.478**	**1**	**<0.001**	**0.000**	**<0.001**	**<0.001**
58	0.065	**0.347**	0.036	-0.124	**0.722**	**0.517**	**0.903**	0.111	**0.697**	**1**	0.335	**<0.001**	**<0.001**
59	0.147	-0.031	**0.847**	**-0.565**	-0.191	-0.314	0.269	**0.521**	**0.585**	-0.166	**1**	0.891	0.891
60	-0.048	0.042	-0.068	-0.106	**0.938**	**0.769**	**0.843**	-0.207	**0.697**	**0.876**	-0.024	**1**	**<0.001**
61	-0.048	0.042	-0.068	-0.106	**0.938**	**0.769**	**0.843**	-0.207	**0.697**	**0.876**	-0.024	**1.000**	**1**

Numbers in the first column correspond to numbers in the first row; 1, overall aroma intensity; 2, gamey aroma; 3, beef-like aroma; 4, metallic aroma; 5, liver-like aroma; 6, herbaceous aroma; 7, off, sour, sweat-like aroma; 8, sweet-associated aroma; 9, overall flavour intensity; 10, gamey flavour; 11, beef-like flavour; 12, metallic flavour; 13, liver-like flavour; 14, herbaceous flavour; 15, off, sour, sweat-like flavour; 16, sweet associated taste; 17, salty taste; 18, sour taste; 19, initial juiciness; 20, sustained juiciness; 21, tenderness; 22, residue; 23, mealiness; 24, Warner-Bratzler shear force; 25, thaw loss percentage; 26, cooking loss percentage; 27, C6:0; 28, C8:0; 29, C10:0; 30, C12:0; 31, C14:0; 32, C15:0; 33, C16:0; 34, C18:0; 35, C20:0; 36, C22:0; 37, C24:0; 38, C14:1; 39, C15:1; 40, C16:1; 41, C17:1; 42, C18:1n9c; 43, C20:1; 44, C18:2n9c; 45, C18:3n6; 46, C18:3n3; 47, C20:2n6; 48, C20:3n6; 49, C20:3n3; 50, C20:5n3; 51, C22:2n6; 52, C22:6n3; 53, total SFA; 54, total MUFA; 55, total PUFA; 56, PUFA:SFA ratio; 57, n6; 58, n3; 59, n6:n3 ratio; 60, total fatty acids; 61, intramuscular fat content. The non-shaded area indicates Pearson correlation coefficients (*r*); area shaded in grey indicates corresponding P-values for Pearson correlation coefficients (*r*).
